# Oral Dysfunction and Cognitive Decline: A Review of Nutritional and Brain Structural Pathways Linking Tooth Loss, Oral Frailty, and Solitary Eating to Dementia

**DOI:** 10.3390/nu18132208

**Published:** 2026-07-07

**Authors:** Hiroyuki Nakamura, Moeko Noguchi-Shinohara, Makoto Murahashi, Kenjiro Ono

**Affiliations:** 1 Department of Oral and Maxillofacial Functional Reconstruction, Graduate School of Medicine, University of the Ryukyus, Okinawa 901-2725, Japan; 2 Department of Oral Surgery, Saiseikai Toyama Hospital, Toyama 931-8533, Japan; 3 Department of Neurology, Graduate School of Medical Sciences, Kanazawa University, Kanazawa 920-8640, Japan; m-nohara@med.kanazawa-u.ac.jp

**Keywords:** tooth loss, oral frailty, cognitive decline, dementia, dietary patterns, brain atrophy, eating alone, nutrition

## Abstract

Dementia is a growing challenge for aging societies, and effective prevention depends on identifying risk factors that can be addressed early, before cognitive decline becomes clinically apparent. Declining oral and dental function, particularly tooth loss, is increasingly recognized as one such factor, and its effect on the brain extends well beyond a reduced ability to chew. This review synthesizes current evidence linking impaired oral function to cognitive decline and dementia, with a focus on nutritional and structural brain pathways. We propose that tooth loss affects the brain through two partly independent routes. The first is nutritional: tooth loss shifts the diet away from fiber- and micronutrient-rich plant foods, which may injure the brain through oxidative stress, inflammation, and gut microbiota changes; the hippocampus, a key memory region, appears especially sensitive to nutritional status. Eating alone adds a social dimension: it is associated with lower diet quality and with reduced volume in memory-related regions, an association only partly explained by diet, since the medial temporal difference persists after dietary adjustment. The second is sensory–neural: the loss of natural teeth removes sensory input from the periodontal ligament and reduces chewing, weakening signaling to the brain and its capacity for plasticity. Importantly, atrophy of the medial temporal lobe and adjacent memory-related regions, together with increased white matter damage, is detectable even in cognitively healthy older adults, and a revised Oral Frailty Five-item Checklist can identify these presymptomatic changes. Because these structural brain changes persist despite denture use and after adjusting for diet, preserving natural teeth may be an especially valuable preventive target. Overall, maintaining oral function is a promising, accessible target for dementia prevention that warrants confirmation in prospective trials.

## 1. Introduction

The global expansion of the aging population has made dementia one of the most pressing public health challenges of the twenty-first century. In Japan, which has one of the world’s most aged societies, dementia is common among older adults, and community-based studies have documented a rising prevalence over recent decades [1,2]; nationwide prospective cohorts have accordingly been established to clarify its risk factors and etiology [3]. Because no curative treatment is currently available, attention has increasingly shifted toward identifying and modifying preventable risk factors during the preclinical stage, when interventions are most likely to alter the disease trajectory.

Against this background, the decline of oral and dental function—particularly tooth loss—has attracted growing attention as a modifiable contributor to cognitive deterioration. Multiple epidemiological studies and meta-analyses have reported that tooth loss is associated with an increased risk of cognitive decline and incident dementia [4,5], and dose–response analyses indicate that the risk rises incrementally as more teeth are lost [6]. The largest synthesis to date, comprising more than four million participants, likewise found tooth loss to be significantly associated with cognitive impairment and all-cause dementia, although its association with Alzheimer’s disease specifically did not reach significance [7]. The precise shape of the relationship, however, remains debated. A meta-analysis of a similarly large population reported no significant association when the number of teeth was modeled as a continuous variable, raising the possibility of a nonlinear relationship; this work has so far been disseminated only as a conference abstract [8] and, pending full peer review, should be regarded as preliminary. Taken together, these observations suggest that clinically meaningful thresholds—rather than incremental tooth count alone—may better capture risk, a view consistent with the severe-tooth-loss (≤9 teeth) threshold that emerges from the structural neuroimaging studies discussed below [9,10]. Moreover, much of the existing literature relies on behavioral or cognitive endpoints, leaving the upstream structural and biological changes that link the oral cavity to the brain comparatively underexplored.

Because the terms used in this field are related but not interchangeable, we distinguish them at the outset. Tooth loss denotes the absence of natural teeth, with edentulism referring to the loss of all teeth; periodontitis is a chronic inflammatory disease of the tooth-supporting tissues that is a leading cause of tooth loss but also exerts systemic inflammatory effects in its own right; reduced masticatory performance refers to impaired chewing, which may result from tooth loss, absent or ill-fitting dentures, or diminished muscular capacity; denture use reflects prosthetic rehabilitation of lost teeth; and oral frailty is a broader, multidimensional age-related decline in oral function that encompasses several of these features together with difficulties in swallowing, articulation, and oral dryness. These exposures overlap but are not equivalent, and we specify which is meant when summarizing individual studies.

A particularly important advance has been the recognition that the influence of impaired oral function extends well beyond the loss of masticatory capacity itself. Tooth loss alters food selection and nutrient intake, shifting dietary patterns toward reduced consumption of plant-based foods and increased intake of energy-dense, processed items [11]. These nutritional changes may affect the brain through oxidative stress, systemic inflammation, and gut-microbiota-mediated pathways [12,13]. In parallel, the social context of eating has emerged as a distinct dimension: solitary eating and social isolation are associated with poorer dietary quality and with structural brain changes, in part independently of nutrition [14,15]. To capture this multidimensional deterioration, the concept of oral frailty has been formalized, and in 2024, three Japanese academic societies jointly proposed the Oral Frailty Five-item Checklist (OF-5) as a standardized, accessible screening tool [16]. Emerging work indicates that oral frailty predicts not only adverse general health outcomes but also incident mild cognitive impairment and presymptomatic brain changes [10,17].

Recent neuroimaging studies suggest that the structural correlates of impaired oral function are detectable even in cognitively unimpaired older adults. Severe tooth loss has been linked to parahippocampal atrophy and increased white matter hyperintensity volume in individuals with normal cognition [9], and dental status has been associated with the longitudinal progression of hippocampal atrophy in community-dwelling cohorts [18]. These observations raise the possibility that oral function may serve as an early, readily assessable marker of brain vulnerability and that its maintenance could represent a viable target for dementia prevention. However, the mechanisms connecting the oral cavity to the brain—spanning reduced sensory input from the periodontal ligament, chronic systemic inflammation, and nutritional and social pathways—remain incompletely integrated, and their relative contributions are still debated.

**Figure 1:**
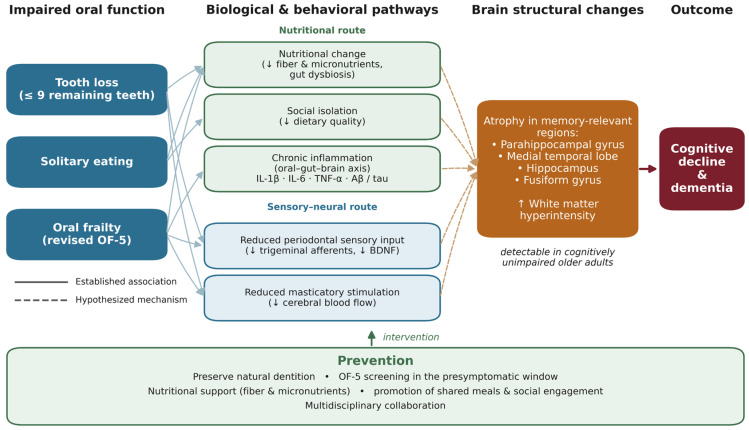
Conceptual overview of the pathways linking impaired oral function to cognitive decline and dementia. Impaired oral function—comprising tooth loss, oral frailty, and solitary eating—influences the brain through interacting nutritional, social, inflammatory, sensory, and masticatory pathways that converge on the atrophy of memory-relevant regions and accumulation of white matter injury, changes that are detectable even in cognitively unimpaired older adults. Preventive intervention acts across the cascade. Pathway boxes are shaded by route (green, nutritional; blue, sensory–neural). Solid arrows denote associations supported by human epidemiological or neuroimaging data, whereas dashed arrows denote hypothesized mechanistic links that remain to be confirmed. BDNF, brain-derived neurotrophic factor; IL, interleukin; OF-5, Oral Frailty Five-item Checklist; TNF, tumor necrosis factor; Aβ, amyloid-β.

This review synthesizes the current evidence linking oral and dental dysfunction to cognitive decline and dementia, with particular emphasis on nutritional and structural brain pathways relevant to a nutrition-focused readership. We first examine how tooth loss and solitary eating reshape dietary and nutritional status. We then summarize the structural brain changes associated with tooth loss and oral frailty, including the development and validation of the revised OF-5. Next, we review the biological mechanisms—sensory, inflammatory, and masticatory—through which oral dysfunction may influence brain health. Finally, we discuss preventive strategies and future directions, with the aim of positioning the maintenance of oral function as an integral component of comprehensive dementia-prevention efforts. An overview of the conceptual framework adopted throughout this review is presented in Figure 1.

Although this is a narrative rather than a systematic review, source identification followed a structured approach. We searched PubMed and Google Scholar for English-language articles published up to June 2026, combining terms related to oral function (e.g., “tooth loss,” “oral frailty,” “mastication,” “denture,” “periodontitis”) with terms related to nutrition, brain structure, cognition, and dementia (e.g., “nutrient intake,” “dietary pattern,” “hippocampus,” “white matter hyperintensity,” “cognitive decline,” “dementia”). Priority was given to peer-reviewed epidemiological, neuroimaging, and meta-analytic studies, supplemented by mechanistic and experimental work where relevant to pathway interpretation, and the reference lists of key articles were screened for additional sources. Because the synthesis is narrative, study selection was purposive rather than exhaustive, and no formal risk-of-bias assessment was undertaken.

## 2. Dietary and Nutritional Changes Associated with Tooth Loss

One of the most direct consequences of tooth loss is a change in what older adults are able and choose to eat. Because mastication governs the range of foods that can be comfortably consumed, the loss of teeth reshapes both food selection and the resulting nutrient profile. In a population-based study of cognitively normal older adults, individuals with severe tooth loss (nine or fewer remaining teeth) showed significantly reduced intake of plant-based foods together with increased consumption of fats and processed items [9]. At the nutrient level, this manifested as lower intake of dietary fiber, manganese, copper, iron, vitamin K, and vitamin C, alongside a relative increase in total energy intake. Such a shift—away from micronutrient-dense plant foods and toward energy-dense alternatives—mirrors the broader “Western” dietary pattern linked to long-term cognitive decline and adverse brain changes [12].

Beyond individual nutrients, the pattern of food intake associated with tooth loss appears to carry cognitive relevance. Using reduced rank regression to derive dietary patterns across the whole diet, a characteristic tooth loss-related pattern was identified, marked by decreased consumption of pickled vegetables, lettuce and cabbage, green leafy vegetables, and carrots and squash, with a compensatory increase in white rice intake [11]. Importantly, older adults whose diets most closely matched this pattern were at a considerably higher risk of cognitive impairment. This finding is consistent with systematic reviews of neuroimaging studies, in which healthier, anti-inflammatory dietary patterns are associated with preserved brain structure, whereas proinflammatory patterns track with markers of neurodegeneration and silent vascular injury [13]. Large-cohort evidence is concordant: among 8938 older Japanese adults in the Japan Prospective Studies Collaboration for Aging and Dementia (JPSC-AD), a traditional Japanese dietary pattern rich in protein and minerals was associated with a lower prevalence of dementia and with reduced white matter lesion volume [19].

The social context in which meals are eaten represents a further influence on dietary quality and brain structure, one that is only partly mediated by nutrition. In a study of 727 cognitively unimpaired older Japanese adults, those who habitually ate alone demonstrated a less healthy dietary profile, with lower protein intake, a higher carbohydrate-to-energy ratio, greater alcohol consumption, and reduced intake of polyunsaturated fatty acids compared with those who ate with others [14]. These solitary eaters also exhibited smaller volumes in brain regions critical for cognition, particularly the medial temporal lobe (MTL) and hippocampus. Notably, the difference in hippocampal volume between solitary and social eaters was attenuated after adjustment for dietary factors, suggesting that the hippocampal effect is largely nutritionally mediated; in contrast, the MTL difference persisted, pointing to additional, non-nutritional contributions, such as reduced social interaction. Importantly, the influence of solitary eating is unlikely to be confined to diet: eating alone commonly accompanies loneliness, depressive symptoms, bereavement, functional decline, and reduced social and cognitive stimulation, each of which may independently contribute to cognitive risk. Eating alone is therefore not merely a marker of social isolation but a behavior with measurable nutritional and structural correlates that also indexes a broader psychosocial vulnerability.

How might these dietary shifts translate into brain vulnerability? Several converging pathways have been proposed. Reduced intake of vegetables and other antioxidant-rich foods may increase oxidative stress and promote neuronal injury, while diminished dietary fiber can alter the gut microbiota in ways that affect systemic and central inflammation [12]. Experimental evidence supports a causal role for the latter: in mice, a fiber-deprived diet induced gut microbial dysbiosis and microbial metabolite changes that drove hippocampal microglia-mediated synaptic loss and cognitive impairment [20]. Circulating nutritional markers may capture the downstream consequences of these processes. In a large cohort of cognitively normal community-dwelling older adults from the JPSC-AD, lower serum albumin—reflecting nutritional status as well as antioxidant and anti-inflammatory capacity—was associated with smaller total brain and hippocampal volumes, independent of physical frailty [21]. Notably, among the brain regions examined across these studies, hippocampal volume appears to be the most consistently nutrition-linked: in a community-based longitudinal study, greater adherence to a Western dietary pattern predicted smaller left hippocampal volume over a four-year interval [22], the solitary-eating-related hippocampal difference was largely attenuated after dietary adjustment [14], and lower hippocampal volume was independently associated with serum albumin, a circulating nutritional marker [21]—though nutrition is best regarded as one important modifiable contributor rather than the sole determinant of hippocampal integrity. Together, these observations suggest that the nutritional sequelae of tooth loss act on the brain through oxidative, inflammatory, and microbiota-related mechanisms rather than through caloric deprivation alone.

The therapeutic corollary—that improving diet might preserve cognition—remains, however, only partially substantiated. A randomized controlled trial of the Mediterranean–DASH Intervention for Neurodegenerative Delay (MIND) diet found no significant cognitive benefit over a control diet after 3 years of intervention in older adults [23]. This neutral result tempers expectations that dietary modification alone can reverse established risk and highlights an important nuance for the present discussion: if the dietary changes accompanying tooth loss are downstream of an underlying loss of oral function, then nutritional counseling may be insufficient unless the upstream oral deficit is also addressed. This possibility motivates the examination, in the following sections, of the structural brain changes and biological mechanisms that connect oral dysfunction to cognitive decline.

## 3. Brain Structural Changes Associated with Tooth Loss and Oral Frailty

Perhaps the most consequential development in this field has been the demonstration that the brain correlates of impaired oral function are detectable well before any clinical cognitive decline. In a population-based study of cognitively normal older adults, severe tooth loss (nine or fewer remaining teeth) was associated with atrophy of the parahippocampal gyrus and an increased volume of white matter hyperintensities (WMH) [9]. Both of these features are characteristically observed in patients with dementia, yet they were present in individuals whose cognition remained intact. Longitudinal data reinforce this cross-sectional picture: in the community-dwelling Ohasama cohort, dental status was associated with the rate of hippocampal atrophy over a 4-year interval, with fewer teeth predicting faster left hippocampal volume loss among individuals with mild periodontitis [18]. Taken together, these findings position tooth loss as a marker of incipient, subclinical neurodegeneration rather than merely a comorbidity of established disease. Table 1 summarizes the principal human studies linking impaired oral function to brain structural and cognitive outcomes.

Whether prosthetic rehabilitation can offset these structural changes is an important and clinically contested question, and the evidence is nuanced. In cognitively normal older adults with severe tooth loss, parahippocampal atrophy and increased WMH were observed regardless of whether dentures were used, suggesting that denture-based functional restoration does not fully compensate for the loss of natural teeth [9]. A plausible explanation lies in the unique sensory role of the periodontal ligament: when natural teeth and osseointegrated implants were compared, tactile stimulation of natural teeth elicited greater cerebral cortical activation, indicating that the rich mechanoreceptive input from the periodontal ligament cannot be wholly reproduced by artificial replacements [27]. This does not mean that dentures are without value; on the contrary, in a 10-year prospective cohort of older adults with partial tooth loss, denture use was associated with better baseline cognition and a slower rate of cognitive decline [24]. The two observations can be reconciled: dentures partially restore masticatory function and may slow cognitive decline, yet they cannot recreate the natural sensory afferents whose loss is mirrored in the persistent structural changes. The degree of benefit may also depend on the pattern of tooth loss. In partial tooth loss—where natural teeth with an intact periodontal ligament remain but have lost their opposing (antagonist) teeth—a denture that re-establishes occlusal contact can restore masticatory loading, and thereby periodontal mechanoreceptor stimulation, to those remaining natural teeth; this may help explain why denture use was associated with slower cognitive decline in a cohort characterized by partial tooth loss [24]. Where teeth are absent altogether, by contrast, the periodontal ligament itself is gone, and no prosthesis can supply afferent input from the edentulous region. Although the clinical magnitude of this sensory difference and its cognitive consequences remains uncertain, this distinction highlights the likely importance of preserving natural dentition wherever possible.

These observations also motivated a shift in emphasis from tooth count alone toward a broader, functional construct. In 2024, three Japanese academic societies—the Japan Geriatrics Society, the Japanese Society of Gerodontology, and the Japanese Association on Sarcopenia and Frailty—jointly issued a consensus statement standardizing the assessment of oral frailty through the OF-5 [16]. The OF-5 comprises five readily assessed components: fewer remaining teeth, difficulty chewing, difficulty swallowing, dry mouth, and reduced articulatory oral motor skill, with oral frailty defined as the presence of at least two. This tool has prognostic validity beyond the oral cavity: oral frailty defined by the OF-5 predicts physical disability and mortality in community-dwelling older adults [28], and, of particular relevance here, it is associated with an increased risk of incident mild cognitive impairment (MCI) [17].

Because the standardized OF-5 sets its tooth-count criterion at 19 or fewer teeth, it may not capture severe tooth loss (nine or fewer teeth) related to brain atrophy. To address this, a revised OF-5 in which the tooth-count criterion is tightened from 19 or fewer to nine or fewer remaining teeth was developed and validated in cognitively unimpaired older adults [10]. The components of the standard and revised OF-5 are summarized in Table 2.

The added criterion materially improved the sensitivity. Whereas the original OF-5 detected only reduced fusiform gyrus volume, the revised version identified a broader constellation of dementia-related changes: higher WMH volume together with smaller volumes of the MTL, pars triangularis, and fusiform gyrus [10]. Critically, these associations persisted after adjustment for nutrient intake and food consumption, indicating that the brain changes captured by oral frailty are not explained by nutrition alone. The affected regions are functionally significant; degenerative changes in the fusiform gyrus have been reported in patients with amnestic MCI [29], and medial temporal and parietal atrophy is closely tied to impaired cognitive function in dementia [30].

Collectively, these structural studies converge on a coherent narrative. Impaired oral function—whether quantified as severe tooth loss or multidimensional oral frailty—is accompanied by atrophy in memory-relevant brain regions and accumulation of white matter injury. These changes are demonstrable in cognitively healthy individuals and only partially reversible through prosthetic means. A simple, noninvasive checklist, such as the revised OF-5, may therefore serve as an early flag for brain vulnerability, providing a practical entry point for preventive intervention. The biological routes through which oral dysfunction produces these changes are considered next.

## 4. Biological Mechanisms

The structural and epidemiologic associations described above are underpinned by several biological pathways through which impaired oral function may influence brain health. These mechanisms are not mutually exclusive; rather, they operate in parallel and interact, encompassing chronic systemic and central inflammation and the loss of sensory and masticatory input from the dentition. Each is considered in turn below.

### 4.1. Chronic Inflammation and the Oral–Gut–Brain Axis

One extensively studied pathway involves chronic inflammation originating in the diseased periodontium. Periodontal disease drives both local and systemic inflammatory responses, and the resulting low-grade systemic inflammation links periodontitis to a range of inflammatory comorbidities [31]. Periodontal pathogens and their metabolites can enter the circulation and elevate systemic levels of proinflammatory cytokines, such as interleukin (IL)-1β, IL-6, and tumor necrosis factor (TNF)-α [32,33]. These mediators are capable of provoking neuroinflammation and may thereby accelerate brain aging and promote the accumulation of amyloid-β and tau pathology. Human data increasingly substantiate this chain: in older adults, periodontitis was associated with poorer cognitive performance and a higher risk of cognitive progression, and blood-based biomarkers of Alzheimer’s disease contributed to this relationship [25]. Neuroimaging evidence is concordant; in an elderly cohort, the clinical, microbiological, and serological features of periodontitis were associated with magnetic resonance imaging markers related to the risk of Alzheimer’s disease and related dementias (ADRD) [26]. Beyond the periodontal pathogens themselves, a broader disturbance of the oral microbial community appears relevant: a systematic review found that oral microbiota dysbiosis is associated with an increased risk of dementia [34]. This last observation dovetails with the nutritional pathway discussed earlier, since reduced dietary fiber intake following tooth loss can alter gut microbial composition and promote neuroinflammation [20], suggesting that the oral and gut microbiomes may act in concert along a shared oral–gut–brain axis. More broadly, gut microbial dysbiosis and its attendant inflammatory and short-chain fatty-acid-mediated signaling have been implicated in the pathogenesis of Alzheimer’s disease, prompting interest in dietary, prebiotic, and probiotic strategies that target this axis [35].

### 4.2. Reduced Sensory and Masticatory Input

A second pathway concerns the loss of sensory and masticatory input that the teeth themselves supply to the brain. The periodontal ligament that anchors each natural tooth is densely populated with mechanoreceptors, which sense tooth load and contact and relay this information to the brain through the trigeminal nerve. The resulting stream of afferent signals is thought to help sustain neural plasticity. Its richness is striking: in conscious macaque monkeys, periodontal mechanoreceptive neurons projecting to the somatosensory cortex display remarkably complex receptive fields [36]. When teeth are lost, this input falls away—and the consequences reach memory-relevant brain regions.

Evidence for this link spans animal and human studies. In mice, removing the molars lowered hippocampal levels of brain-derived neurotrophic factor (BDNF) and impaired spatial memory; notably, environmental enrichment reversed these deficits [37]. Direct human evidence was scarce until recently, but it is now accumulating—much of it from trigeminal neuralgia, a disorder marked by chronic disturbance of trigeminal sensory processing. Patients with this condition show poorer cognitive performance alongside altered resting-state activity in the temporal pole, superior temporal gyrus, and insula [38], and structural imaging points to the very region affected by tooth loss: gray-matter volume in the parahippocampus and temporal lobe shrinks in proportion to disease duration [39]. Together, these findings indicate that disrupted oral afferent input is mirrored by changes in the medial temporal lobe.

Two practical implications follow for prosthetic treatment. First, artificial replacements cannot fully restore the natural sensory signal: tactile stimulation of natural teeth evokes greater cortical activation than stimulation of implants [27], because the mechanoreceptive contribution of the periodontal ligament is not reproducible by prosthetic means. Second, dentures nonetheless retain real value. Beyond restoring chewing—the component of oral function most amenable to prosthetic repair, consistent with evidence that denture use can slow cognitive decline [24]—they re-establish occlusal contacts. By doing so, a denture restores masticatory loading, and hence periodontal mechanoreceptor stimulation, of the remaining natural teeth that still possess a periodontal ligament. What it cannot do is supply such afferents from the edentulous region itself, where the ligament has been lost along with the teeth.

### 4.3. Integration of Pathways

These mechanisms are deeply interconnected. Chronic inflammation can suppress the production of neurotrophic factors, such as BDNF, compounding the plasticity deficits caused by reduced sensory and masticatory input. Conversely, the diminished afferent signaling that accompanies tooth loss may lower the threshold at which inflammatory and vascular insults translate into structural injury. Superimposed on these are oxidative stress and impaired vascular function, which the nutritional changes described earlier are well positioned to aggravate. At a broader level, these pathways can be grouped into two partly independent routes through which tooth loss affects the brain: a nutritional route, operating through diet-related changes in nutrient intake, inflammation, and the microbiota, and a sensory–neural route, operating through the loss of periodontal mechanoreceptor input and reduced masticatory stimulation. That the two are dissociable is underscored by evidence that oral frailty-related brain changes persist after adjustment for nutrient intake and food consumption [10], and structural changes are present irrespective of denture use [9], pointing to a sensory–neural contribution that neither dietary nor purely functional (chewing) restoration can fully address. The convergence of these inflammatory, sensory, masticatory, nutritional, and microbiota-related pathways on the same memory-relevant brain regions offers a coherent explanation for why impaired oral function is associated with early neurodegenerative change and why effective prevention is likely to require strategies that address several of these pathways simultaneously.

## 5. Prevention Strategies and Future Perspectives

The evidence reviewed in the preceding sections carries a clear practical implication: because the structural brain changes associated with impaired oral function are detectable while cognition is still intact [9,18], a window opens during which preventive intervention may be especially valuable. It is useful here to distinguish three related but separate goals: reducing the incidence of dementia, slowing the rate of cognitive decline, and identifying presymptomatic brain vulnerability so that at-risk individuals can be flagged early. The evidence reviewed here speaks most directly to the last of these, and any preventive claim should be framed accordingly. A central component of any such strategy is likely to be the preservation of natural teeth through regular dental examination and early treatment, since the sensory afferents of the periodontal ligament cannot be fully reproduced by prosthetic means [27]. Where tooth loss has already occurred, appropriate prosthetic rehabilitation remains worthwhile, as denture use is associated with better cognition and a slower rate of decline [24]—though the magnitude of any such benefit remains uncertain—and it is best regarded as complementary to, rather than a substitute for, the maintenance of natural dentition.

Building on this foundation, a staged, individualized program can be envisioned. The first stage is systematic screening of oral function. A simple, noninvasive instrument such as the revised OF-5—which can be completed without a dental professional—offers a practical means of identifying at-risk individuals early, given its demonstrated associations with incident mild cognitive impairment and presymptomatic brain changes [10,17]. The second stage is targeted restoration of oral function, delivered through professional oral care and, where indicated, training in chewing and swallowing. The third stage integrates nutritional and social support: because the nutritional sequelae of tooth loss act on the brain through inflammatory and microbiota-related routes, dietary counseling aimed at restoring micronutrient-dense, fiber-rich intake is a logical component, complemented by circulating markers of nutritional status, such as serum albumin, that track with brain volume in cognitively normal adults [21].

Importantly, the social dimension of eating should be addressed alongside its nutritional content. Solitary eating is linked to reduced brain volume in part independently of diet [14], and lower frequency of social contact and greater social isolation are themselves associated with brain atrophy and cognitive decline across diverse populations [40,41]. Interventions that promote shared meals and community engagement target a pathway that nutrition alone cannot reach. Delivering this multidimensional program requires coordinated, multidisciplinary collaboration—linking dental professionals, dietitians, rehabilitation specialists, and community support services—so that screening, oral-function restoration, nutritional guidance, and social support are provided in an integrated rather than fragmented manner.

Several priorities should guide future research. First, and most importantly, the central hypothesis that improving oral function prevents or delays cognitive decline must be tested in adequately powered, prospective intervention trials; the largely neutral result of a major dietary-intervention trial [23] suggests that addressing nutrition in isolation may be insufficient and reinforces the rationale for trials that target the upstream oral deficit directly. Second, the biological mechanisms linking oral dysfunction to neurodegeneration—particularly the dynamics of inflammatory mediators and their effects on neuroplasticity and the interplay of the oral and gut microbiomes [34]—warrant a more detailed mechanistic investigation. Third, comprehensive preventive strategies should incorporate social determinants and should be evaluated for generalizability beyond the predominantly Japanese cohorts in which much of the current evidence has been generated. Alongside these scientific questions, structural measures, such as improving older adults’ access to oral health care and strengthening community food-support systems, represent important complementary goals.

Finally, these scientific developments are increasingly reflected in public health policy. The inclusion of a universal dental health checkup program in Japan’s 2022 national policy framework illustrates a growing recognition that maintaining oral health from the presymptomatic stage may contribute to dementia prevention. Realizing this potential will depend on translating the evidence summarized here into accessible, equitable, and lifelong programs of oral-function maintenance.

## 6. Limitations of the Evidence

Several limitations of the current evidence base should temper the interpretation of this review. First, most of the human data are observational and cross-sectional, so the associations described cannot establish causality, and reverse causation—whereby incipient neurodegeneration impairs self-care and accelerates tooth loss—remains difficult to exclude. Intervention data are correspondingly sparse: no adequately powered randomized trial has yet shown that improving oral function reduces the incidence of cognitive decline or dementia, and the one major dietary-intervention trial discussed above was essentially neutral [23].

Second, the constructs discussed here—tooth loss, periodontitis, oral frailty, denture use, edentulism, and reduced masticatory performance—overlap but are not equivalent exposures, and their definitions and measurement vary considerably across studies. This heterogeneity limits direct comparison between studies and the precision with which any single exposure can be linked to a specific brain change.

Third, residual confounding is an important concern. The authors’ own cohort analyses have attempted to address this in part: the parahippocampal atrophy and white matter findings were observed irrespective of denture use [9], the oral-frailty-related brain changes persisted after adjustment for nutrient intake and food consumption [10], the solitary-eating-related hippocampal difference was examined with and without adjustment for dietary factors [14], and the serum albumin–brain volume association was independent of physical frailty [21]. Nevertheless, several potentially influential confounders were not fully accounted for and warrant explicit attention in future work, including socioeconomic status and education, depressive symptoms, APOE genotype, smoking, diabetes and broader vascular burden, access to and use of dental care, and pre-morbid dietary habits. Because these factors can independently influence both oral status and cognition, the magnitude of the associations reported here should be regarded as provisional.

Fourth, much of the primary empirical support for the review’s central claims—the tooth-loss-related dietary pattern, the solitary-eating and brain-volume findings, the revised OF-5 validation, the parahippocampal atrophy and white matter data, and the serum albumin–brain volume relationship—is derived from studies conducted by our own group in Japanese cohorts. Several of the strongest claims (for example, parahippocampal atrophy with severe tooth loss and the revised OF-5 predicting presymptomatic brain changes) currently rest on single studies, and independent replication from non-Japanese and non-author-affiliated cohorts remains limited. The evidence base is therefore narrower and less independently generated than the breadth of the topic might suggest.

Fifth, and relatedly, the generalizability of these findings is uncertain. Japan’s dietary norms (the traditional Japanese diet versus Western dietary patterns), its dental-health policies and access to care, and the prevalence and social meaning of solitary eating among older adults may differ substantially from those in other countries and health systems, so the associations reported here may not transfer directly to non-Japanese populations. Finally, some of the biomarkers relied upon are nonspecific; serum albumin, for instance, reflects not only nutritional status but also inflammation, hepatic and renal function, and hydration, and should therefore be interpreted as a general marker rather than a specific index of nutritional adequacy. This nonspecificity argues for structured geriatric nutritional assessment—combining validated instruments such as the Mini Nutritional Assessment and the Geriatric Nutritional Risk Index with a panel of biochemical markers—rather than reliance on any single index, particularly given that malnutrition in older adults frequently coexists with inflammation, sarcopenia, and dysphagia [42].

## 7. Conclusions

Converging evidence indicates that impaired oral and dental function—encompassing tooth loss, oral frailty, and solitary eating—is associated with adverse dietary and nutritional changes and with structural brain alterations in memory-relevant regions, alterations that are already detectable in cognitively unimpaired older adults. These effects appear to be mediated by interacting sensory, inflammatory, masticatory, nutritional, and social pathways rather than by any single mechanism, which helps explain why prosthetic restoration alone appears only partially protective and why preserving natural dentition is likely to be particularly important. The maintenance of oral function, together with the nutritional and social dimensions of eating, represents a plausible and readily accessible target for dementia prevention rather than an established intervention, and simple screening tools offer a practical means of identifying at-risk individuals during the presymptomatic window. Prospective intervention trials that directly target oral function will be needed to establish whether such strategies can ultimately reduce the burden of cognitive decline.

## Figures and Tables

**Table 1:** Summary of principal human studies and meta-analyses linking impaired oral function to brain structural and cognitive outcomes. ^a^ Available only as a conference abstract (not peer-reviewed); included here for completeness because of its scale and contrasting result. MTL, medial temporal lobe; OF-5, Oral Frailty Five-item Checklist; WMH, white matter hyperintensity; MCI, mild cognitive impairment; JPSC-AD, Japan Prospective Studies Collaboration for Aging and Dementia; WHICAP, Washington Heights–Inwood Columbia Aging Project; AD, Alzheimer’s disease; ADRD, Alzheimer’s disease and related dementias; Aβ, amyloid-β.

Study	Design; Country (Cohort)	*N*	Baseline Cognition	Oral Exposure	Principal Finding	Key Adjustments
Nakamura et al. 2024 [9]	Cross-sectional; Japan (Nakajima)	2454	Cognitively normal	Severe tooth loss (≤9 teeth)	Parahippocampal atrophy, ↑ WMH; altered diet	Multivariable-adjusted
Yamaguchi et al. 2023 [18]	4-y longitudinal; Japan (Ohasama)	172	No cognitive decline (≥55 y)	Tooth number × periodontitis	Faster left hippocampal atrophy with fewer teeth in mild periodontitis	Inverse-probability weighting; periodontal depth
Murahashi et al. 2025 [10]	Cross-sectional; Japan (population-based)	732	Cognitively unimpaired	Revised OF-5 (≤9 teeth)	↑ WMH; ↓ MTL, pars triangularis, fusiform	Nutrient intake & food consumption
Isa et al. 2025 [14]	Cross-sectional; Japan	727	Cognitively unimpaired	Solitary eating	↓ MTL & hippocampal volume; poorer diet	Dietary factors
Nagatani et al. 2023 [17]	Prospective; Japan (Kashiwa)	1410	Without cognitive decline	Oral frailty (OF-5)	↑ incident MCI	Multivariable-adjusted
Zhang et al. 2023 [5]	Prospective; UK (UK Biobank)	425,183	Dementia-free	Poor oral health	↑ incident dementia	Multivariable-adjusted
Qi et al. 2024 [24]	10-y prospective; China	27,708	Community-dwelling older adults	Denture use (partial tooth loss)	Slower rate of cognitive decline	Multivariable-adjusted
Usui et al. 2026 [21]	Cross-sectional; Japan (JPSC-AD)	7266	Cognitively normal	Low serum albumin (nutrition)	↓ total brain & hippocampal volume	Physical frailty
Carballo et al. 2023 [25]	2-y prospective; Spain	101	≥60 y, hypertensive	Periodontitis	Poorer cognition; ↑ progression; blood Aβ/tau	Hypertension & covariates
Rubinstein et al. 2024 [26]	Cross-sectional; USA (WHICAP)	468	Elderly	Periodontitis	MRI markers of AD/ADRD risk	Multiple AD/ADRD risk factors
Fu et al. 2024 [7]	Meta-analysis (22 studies); international	4,246,608	Mixed	Periodontal health/tooth loss	↑ cognitive impairment & all-cause dementia (AD not significant)	Study-level adjusted estimates
Chambergo-Michilot et al. 2024 [8] ^a^	Meta-analysis; international	>4,000,000	Mixed	Number of teeth (continuous)	No significant association in continuous model	—

**Table 2:** Components of the standard and revised Oral Frailty Five-item Checklist (OF-5). Oral frailty is operationally defined as the presence of two or more of the five components. In the revised OF-5, the threshold of the tooth-count component (item 1) is changed from ≤19 to ≤9 remaining teeth; this is a modification of item 1 rather than a nested sub-criterion of ≤19 teeth or an additional sixth item. The other four items are unchanged, the instrument remains a five-item checklist, and oral frailty is still defined as two or more positive items [10,16]. To date the revised OF-5 has been validated only in the original Japanese population-based cohort [10]; external validation in independent and non-Japanese populations has not yet been reported.

#	Component	Standard OF-5 Criterion	Revised OF-5 Criterion
1	Number of remaining teeth	≤19 teeth	≤9 teeth (threshold changed from ≤19)
2	Chewing	Difficulty chewing tough foods	Same
3	Swallowing	Difficulty swallowing	Same
4	Oral dryness	Dry mouth	Same
5	Articulatory oral motor skill	Reduced articulatory skill	Same

## Data Availability

Not applicable. No new datasets were generated or analyzed for this review. Data from the primary studies discussed are available as described in their respective original publications.
